# Angiogenesis is inhibitory for mammalian digit regeneration

**DOI:** 10.1002/reg2.24

**Published:** 2014-10-12

**Authors:** Ling Yu, Mingquan Yan, Jennifer Simkin, Paulina D. Ketcham, Eric Leininger, Manjong Han, Ken Muneoka

**Affiliations:** ^1^ Division of Developmental BiologyDepartment of Cell and Molecular BiologyTulane UniversityNew OrleansLA79118USA

**Keywords:** Angiogenesis, blastema, BMP9, digit, mouse, PEDF, regeneration, VEGF

## Abstract

The regenerating mouse digit tip is a unique model for investigating blastema formation and epimorphic regeneration in mammals. The blastema is characteristically avascular and we previously reported that blastema expression of a known anti‐angiogenic factor gene, *Pedf*, correlated with a successful regenerative response (Yu, L., Han, M., Yan, M., Lee, E. C., Lee, J. & Muneoka, K. (2010). BMP signaling induces digit regeneration in neonatal mice. Development, 137, 551–559). Here we show that during regeneration *Vegfa* transcripts are not detected in the blastema but are expressed at the onset of differentiation. Treating the amputation wound with vascular endothelial growth factor enhances angiogenesis but inhibits regeneration. We next tested bone morphogenetic protein 9 (BMP9), another known mediator of angiogenesis, and found that BMP9 is also a potent inhibitor of digit tip regeneration. BMP9 induces *Vegfa* expression in the digit stump suggesting that regenerative failure is mediated by enhanced angiogenesis. Finally, we show that BMP9 inhibition of regeneration is completely rescued by treatment with pigment epithelium‐derived factor. These studies show that precocious angiogenesis is inhibitory for regeneration, and provide compelling evidence that the regulation of angiogenesis is a critical factor in designing therapies aimed at stimulating mammalian regeneration.

## Introduction

In mice the terminal phalangeal bone (P3) is the only limb bone known to have the capacity to regenerate following amputation (Borgens 1982; Neufeld & Zhao 1995; Han et al. 2008; Fernando et al. 2011). In humans the distal region of the fingertip is also reported to have a similar regenerative capacity (Illingworth 1974); thus the rodent digit represents a clinically relevant model for a mammalian regenerative response (Muneoka et al. 2008). Blastema formation associated with this regenerative response is observed following amputation of embryonic, neonatal, and adult digits (Reginelli et al. 1995; Han et al. 2003, 2008; Fernando et al. 2011). In neonates and adults the formation of a proliferative digit blastema follows a complex healing response involving tissue degradation associated with wound closure (Han et al. 2008; Fernando et al. 2011). Blastema cells appear undifferentiated, and current evidence indicates that the cells contributing to the regenerate are lineage restricted (Lehoczky et al. 2011; Wu et al. 2013).

One characteristic of the digit blastema is that it has a reduced number of endothelial cells suggestive of a reduced vasculature (Said et al. 2004; Fernando et al. 2011). In addition, we have recently shown that the regenerative response itself displays dynamic variation in oxygen tension which includes a characteristic hypoxic phase associated with the blastema (Sammarco et al. 2014). Similarly, the blastema formed during limb regeneration in salamanders is reported to have reduced vascularity (Rageh et al. 2002), and it has been suggested that a transient period of avascularity might be critical for the regenerative response (Mescher 1996). We have previously shown that most endothelial cells in the mouse digit blastema express the endothelial cell progenitor marker protein Sca1, suggesting that the regenerating vasculature is derived from a proliferating stem cell population (Fernando et al. 2011). In contrast, the healing of full thickness wounds in mammals is associated with a rapid angiogenic response and the formation of a highly vascularized granulation tissue that undergoes vascular regression during the remodeling phase of the response (Wietecha et al. 2013). The contrasting nature and timing of neovascularization in regeneration versus wound healing suggests that the avascular character of the blastema may be essential for a regenerative response.

The avascular character of the blastema correlates with the expression domain of transcripts for pigment epithelium‐derived factor (PEDF), a factor with potent anti‐angiogenic properties that counteracts vascular endothelial growth factor (VEGF) induced angiogenesis (Muneoka et al. 2008; Liu et al. 2012). PEDF is expressed by a number of different cell types and was first identified as a product of neonatal pigment epithelium cells that induced neuronal differentiation (Steele et al. 1993). Later PEDF was found to be a potent inhibitor of angiogenesis as well as displaying anti‐metastatic and anti‐tumorigenic properties (Becerra & Notario 2013). PEDF inhibits VEGF‐induced angiogenesis of endothelial cells in part by mediating γ‐secretase cleavage of VEGF receptor 1 and inhibiting VEGF induced phosphorylation (Cai et al. 2011). Following digit tip amputation, cells associated with the wound bed express *Pedf* and that expression is maintained during blastema formation (Muneoka et al. 2008). Proximal digit amputations that fail to regenerate do not accumulate *Pedf* expressing cells in the wound bed; however, *Pedf* expression is transiently upregulated in association with digit regenerative responses induced by treatment with bone morphogenetic protein 7 (BMP7) or BMP2 (Yu et al. 2010, 2012). These data are consistent with the idea that neovascular regulation distinguishes a regeneration‐permissive wound environment from wound healing typically associated with scar formation.

In addition to VEGF and PEDF, BMP9 has recently been shown to modulate neovascularization; however, its precise role remains unclear. BMP9 signaling in endothelial cells is mediated by activin receptor‐like kinase 1 (ALK1) and BMP9 functions redundantly with BMP10 (Ricard et al. 2012; Chen et al. 2013). BMP9 is produced in the liver and is present at physiological levels in plasma (Bidart et al. 2012). On the one hand BMP9 is proposed to function as a vascular quiescence factor inhibiting endothelial cell sprouting and counteracting the angiogenic action of VEGF (Scharpfenecker et al. 2007; David et al. 2008; Suzuki et al. 2010). In other models, however, BMP9 is reported to promote endothelial cell proliferation and enhance angiogenesis (Suzuki et al. 2010). In other studies BMP9 induces osteogenic differentiation of mesenchymal stromal progenitor cells in vitro and in vivo (Lamplot et al. 2013) and this osteogenic response is linked to BMP9 induced VEGF expression that is mediated by HIF1α (Hu et al. 2013). Overall, these studies suggest that BMP9 functions in a context‐dependent manner to regulate angiogenesis.

In the current study we have used the mouse neonatal digit tip regeneration model to explore the role that neovascularization plays in mammalian regeneration. During digit tip regeneration we confirm that *Pedf* is expressed in the blastema and early stages of redifferentiation, whereas *Vegfa* transcripts are not detected in the blastema but are expressed during redifferentiation, and *Bmp9* is not expressed at all. Using microcarrier beads we introduced VEGF into the amputation wound to induce precocious angiogenesis and found that VEGF treatment is a potent inhibitor of the regenerative response. In contrast, application of control bovine serum albumin (BSA) treated or PEDF treated beads has no effect on regeneration. These results suggest that precocious angiogenesis of the amputation wound bed is inhibitory for successful regeneration. We next found that BMP9 is also a potent inhibitor of regeneration and that *Vegfa* is upregulated by BMP9. Histological and immunohistochemical analyses of VEGF and BMP9 treatment show that revascularization is enhanced but blastema formation itself is not inhibited, suggesting that the inhibitory action of BMP9 is linked in part to a modification of angiogenesis. Finally, we show that the BMP9 inhibition of regeneration can be rescued by treatment with PEDF, thus demonstrating that a successful regenerative response can be modulated with these extrinsically applied angiogenic modifiers. The evidence suggests that the localized expression of *Pedf* following amputation and during wound healing plays a key role in creating an avascular environment that is permissive for a mammalian regenerative response.

## Results

### VEGF inhibits digit regeneration

**Figure 1:**
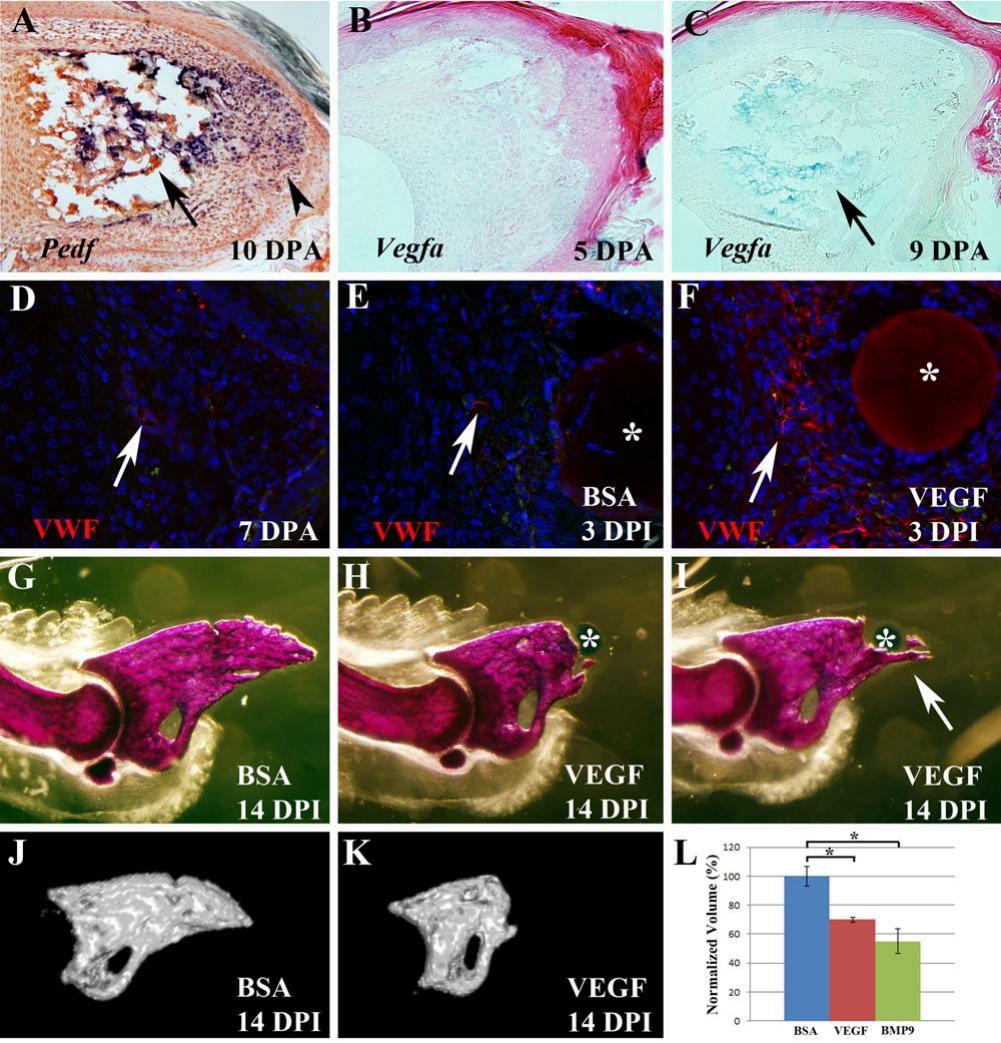
VEGF treatment inhibits digit tip regeneration (top is dorsal and proximal is to the left). (A) At 10 DPA when redifferentiation has initiated, transcripts for the anti‐angiogenic factor *Pedf* are abundant in both the digit stump (arrow) and blastema (arrowhead). (B) During wound healing *Vegfa* expression is not detected in either the stump or wound bed at 5 DPA. (C) At 9 DPA, transcripts for *Vegfa* can be detected in the digit stump (arrow) but not the blastema. (D) Immunohistochemical staining for von Willebrand factor (VWF, red) at 7 DPA showing endothelial cells (arrow) in the blastema. (E) VWF staining of a control sample treated with a BSA bead (*) showing endothelial cells (arrow) in the blastema. (F) VWF staining of an experimental digit treated with a VEGF bead (*) showing an enhanced population of endothelial cells (arrow) associated with the bead. Sections shown in (D)−(F) were counterstained with DAPI. (G) Alizarin Red whole mount staining of 14 DPI digits showing that regeneration is not altered by control BSA treated bead implantation. (H) Based on whole mount staining the majority of digits fail to regenerate following treatment with a VEGF microcarrier bead (*). (I) Some VEGF bead treated digits form partial regenerates that include bony spikes that extend distally from the digit stump (arrow). (J) MicroCT rendering of the P3 skeletal element shown in (G). (K) MicroCT rendering of the P3 element shown in (H). (L) Graph showing normalized volume measurements from microCT analyses of control BSA treated (blue), VEGF treated (red) and BMP9 treated (green) digits at 14 DPI.

To investigate angiogenesis in neonatal digit regeneration we focused on the spatial expression pattern of *Pedf* and *Vegfa* during different stages of regeneration. We had previously found that *Pedf* is prominently expressed during wound healing and blastema formation (4–6 Days Post‐Amputation, DPA) (Muneoka et al. 2008), and we show here that transcripts for *Pedf* remain highly expressed at 10–11 DPA (Fig. 1A) when the stump and proximal regenerate are undergoing osteogenesis (Han et al. 2008). During wound healing and blastema formation *Vegfa* expression is not detected by in situ hybridization (Fig. 1B), and *Vegfa* transcripts are first detected in the stump during stages of redifferentiation (Fig. 1C). We investigated a related angiogenic factor, *Vegfb*, but failed to observe expression at these stages of regeneration (not shown). These results suggest that the early stages of regeneration are associated with an anti‐angiogenic environment and are consistent with the reduced endothelial cell compartment observed in the blastema (Fernando et al. 2011). We also investigated expression of *Bmp9* in the neonatal digit during regeneration and found no evidence of expression during the regenerative response (not shown). This was not surprising since BMP9 is produced by the liver and known to be present in plasma at physiological concentrations (Bidart et al. 2012). These data suggest that angiogenesis is largely inhibited during early stages of digit tip regeneration.

To explore the role that VEGF plays in revascularization we used microcarrier beads to treat amputated digits with purified recombinant VEGF after wound closure (4 DPA) and analyzed regenerates 3 days later (3 days post‐bead implantation, DPI) using the endothelial marker VWF, which detects the von Willebrand factor/factor 8 complex. VWF staining of control blastema sections at a similar stage identified few endothelial cells scattered throughout the blastema (Fig. 1D) consistent with previous findings of adult blastema using the endothelial marker CD31 (Fernando et al. 2011). In regenerates that received a control BSA treated bead implant we observed a similar level of VWF staining (Fig. 1E), indicating that bead implantation did not induce a neovascular response. In contrast, regenerates that received a VEGF treated bead displayed a high level of VWF expression in cells surrounding the bead (Fig. 1F) indicating an enhancement of the endothelial cell population within the blastema.

To test the role of angiogenesis during regeneration we used microcarrier beads to treat amputated digits with purified recombinant PEDF or VEGF after wound closure (4 DPA), analyzing regenerates at 14 DPI. The resulting digits were analyzed as Alizarin Red stained whole mount skeletal preparations and the regeneration response was secondarily analyzed using micro computed tomography (microCT) to quantitate bone volume. MicroCT was also used to generate three‐dimensional renderings of the P3 skeletal element. We have used this targeted bead implantation technique in previous experiments to study BMP signaling during regeneration (Yu et al. 2010). As in previous studies, control BSA treated beads had no effect on the regenerative response (Fig. 1G). Similarly, treatment with PEDF (1000 ng/μL) did not influence the regenerative response (Fig. S1A), but this was expected since *Pedf* is normally expressed at a high level during wound healing and blastema stages. On the other hand, VEGF (500 ng/μL) treatment resulted in the complete inhibition of regeneration in the majority of digits analyzed (Fig. 1H). Based on gross observation of whole mount stained digits, regeneration was inhibited in 24 of 28 digits (86%) with four digits regenerating normally. The majority of inhibited digits formed simple truncations whereas some of the digits (9/28) formed irregular bony outgrowths that appeared to be partial regenerates (Fig. 1I). Bone volume analyses of VEGF treated P3 elements compared with BSA treated controls (Fig. 1J,K) showed a statistically significant reduction following VEGF treatment (Fig. 1L; *P* < 0.01). We next analyzed VEGF treated digits 4–6 weeks after treatment to determine whether the treatment simply delayed the regenerative response and found that the inhibited digit phenotype was maintained (*n* = 28; Fig. S1B). We addressed the potency of the VEGF effect by testing different bead soaking concentrations (Fig. S1C). We found 83% inhibition at 100 ng/μL (*n* = 18), 23% inhibition at 50 ng/μL (*n* = 22) and no inhibitory effect at 10 ng/μL (*n* = 18). These results show that administration of VEGF following digit amputation effectively inhibits the regenerative response in a dose‐dependent manner.

### BMP9 inhibits digit regeneration

**Figure 2:**
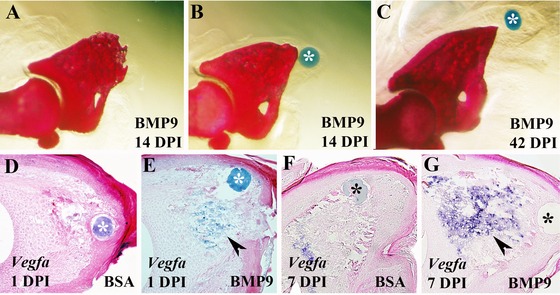
BMP9 treatment inhibits digit tip regeneration (top is dorsal and proximal is to the left). (A), (B) Alizarin Red whole mount stained digit at 14 DPI treated with a BMP9 microcarrier bead (*). Truncated digit tips formed in approximately half of the BMP9 treated digits (A), whereas the remaining digits formed a shortened but anatomically distinct digit tip suggestive of a remodeling response (B). (C) The majority of BMP9 treated digit tips analyzed at 42 DPI formed shortened digit tips. (D)−(G) Section in situ hybridization studies to localize *Vegfa* transcripts after treatment with a control BSA or a BMP9 microcarrier bead. (D) *Vegfa* expression was not detected at 1 DPI in BSA treated control digits. (E) Following BMP9 treatment *Vegfa* transcripts were abundant in the digit stump (arrowhead) but not directly associated with the microcarrier bead (*). (F) At 7 DPI *Vegfa* expression was detected in BSA treated control digits at a level similar to that of untreated regenerates at a similar stage (see Fig. 1C). (G) *Vegfa* expression remained upregulated (arrowhead) in the digit stump and proximal blastema 7 days after treatment with BMP9.

BMP9 is a systemically available factor that functions in regulating vascular structure postnatally (Ricard et al. 2012; Chen et al. 2013) and has been shown to be either pro‐angiogenic or anti‐angiogenic in different assays (Scharpfenecker et al. 2007; David et al. 2008; Suzuki et al. 2010). While *Bmp9* transcripts are not detected during digit regeneration, treating amputated digits with BMP9 (500 ng/μL) resulted in the complete inhibition of regeneration (Fig. 2A). Analysis of BMP9 treated digits showed that 100% of the sample digits analyzed at 14 DPI displayed an inhibited regenerative response based on gross inspection (*n* = 26). Bone volume analyses of BMP9 treated digits resulted in a significant reduction in final P3 volume compared with BSA controls (Fig. 1L; *P* < 0.01). The bone volume of BMP9 treated digits was not significantly different compared with VEGF treatment. We did note some qualitative differences between BMP9 and VEGF inhibited digits. While VEGF treatment results in truncated digit stumps, we observed two distinct phenotypes in BMP9 inhibited samples: (1) truncated digit stumps that lacked the digit tip (12/26; Fig. 2A) and (2) shortened digits in which the digit stump appeared to be remodeled to form a shortened but anatomically distinct tapered digit tip (14/26; Fig. 2B). We next analyzed BMP9 treated digits 6 weeks after treatment to determine whether the treatment delayed the regenerative response and found that all digits were inhibited (*n* = 12) but that the majority (11/12) displayed the remodeled phenotype (Fig. 2C). We addressed the potency of the BMP9 effect by testing bead soaking concentrations of 100, 50 and 10 ng/μL (Fig. S1D) and found that all digits were inhibited at 100 (*n* = 23) and 50 ng/μL (*n* = 26) but all digits regenerated normally at 10 ng/μL (*n* = 35). These results indicate that BMP9 is an effective and potent inhibitor of endogenous digit tip regeneration.

Since both VEGF and BMP9 are known regulators of angiogenesis and both inhibit the regeneration response, we investigated the possibility that the two inhibitory activities might be related. *Bmp9* is not expressed during the regeneration response so we focused our studies on the possibility that BMP9 influenced the expression of *Vegfa*. We carried out a microarray analysis of digit amputations 24 h following treatment with either BMP9 or BSA. For this analysis we collected microdissected wound mesenchymal tissue associated with the microcarrier bead to enrich for cells directly influenced by BMP9 (Fig. S2A). Genes that were significantly upregulated (181) or downregulated (67) are listed in Table 1 (*P* < 0.005; fold change > 2). The microarray data were validated using real‐time reverse transcription polymerase chain reaction (RT‐PCR) targeting five genes from this list (Fig. S2B). The microarray analysis identified a total of 10 genes with known association to angiogenesis that were significantly changed (Table 1). Of these, nine were upregulated (*Arhgap29*, *Cdh13*, *Chrdl1*, *Fgf18*, *Fgfr3*, *Ptx3*, *Smoc2*, *Vegfa*, *Vwf*) and one was downregulated (*Ndp*). Since *Vegfa* was among the upregulated genes we explored changes in expression based on in situ hybridization 24 h after BMP9 treatment. *Vegfa* transcripts were not detected in control digits (Fig. 2D), whereas expression was induced in a population of cells in the distal digit stump following BMP9 treatment (Fig. 2E). Seven days following treatment with BSA, we found levels of *Vegfa* expression in the stump (Fig. 2F) that were comparable to unmanipulated regenerates (Fig. 1C). On the other hand, 7 days following BMP9 treatment a high level of *Vegfa* expression was observed throughout the stump and proximal blastema (Fig. 2G). We note that *Vegfa* expression was not induced in cells directly surrounding the BMP9 bead suggesting that BMP9 is acting in a cell‐type specific manner to induce *Vegfa*. These data show that BMP9 induced a rapid and sustained upregulation of *Vegfa* expression in the regenerating digits and, together with the demonstration that VEGF treatment inhibits regeneration, suggest that BMP9 inhibited regeneration was mediated by VEGF.

**Table 1:** Microarray analysis of BMP9 treatment at 24 h

Upregulated*P* < 0.005FC > 2.0*n* = 181Bold, vascular	*1600014C10Rik*, *2310034C09Rik*, *8430408G22Rik*, *Acan*, *Acsl3*, *Adcy2*, *Amtn*, *Appl2*, *Arc*, ***Arhgap29*** ^1^, *Arhgef28*, *Asic3*, *Atg4b*, *Atoh8*, *AW125324*, *C2cd2*, *Calcoco1*, *Car12*, *Cbr2*, *Ccbp2*, *Ccdc136*, ***Cdh13*** ^2^, *Ces2f*, *Chdh*, ***Chrdl1*** ^3^, *Chrdl2*, *Chst8*, *Cited1*, *Cited4*, *Cldn3*, *Clk3*, *Col4a5*, *Col9a3*, *Colec12*, *Cpm*, *Crebl2*, *Cryab*, *Ctnnd2*, *Cux1*, *Cybrd1*, *Cyp26a1*, *D16Ertd472e*, *Dclk1*, *Dmpk*, *Dnajb2*, *Dnase1l2*, *Dsg4*, *Dusp10*, *Dusp14*, *Dyrk2*, *Ehd3*, *En1*, *Enpp2*, *Fabp7*, *Fam129a*, *Fam132a*, *Fam19a5*, *Fam214a*, *Fam214b*, *Fam49a*, *Fbln7*, *Fbxo36*, ***Fgf18*** ^4^, ***Fgfr3*** ^4^, *Fgfrl1*, *Fkbp11*, *Fmod*, *Foxn1*, *Foxp1*, *Foxq1*, *Frzb*, *Fxyd4*, *Fyn*, *Fzd9*, *Gdf10*, *Gdpd5*, *Gldn*, *Gm829*, *Gpr125*, *Gpr68*, *Gprc5d*, *Grem2*, *Gsdma*, *H19*, *Hey1*, *Hipk3*, *Hk2*, *Ihh*, *Inadl*, *Ing2*, *Islr*, *Jmy*, *Klhl13*, *Kprp*, *Krt33a*, *Krt81*, *Krtap11–1*, *Krtap13*, *Lgr6*, *Lman1l*, *Lnx1*, *Lpin3*, *Lrrc15*, *Lrrc20*, *Lrrc4b*, *Lyg1*, *Mapre3*, *Mcoln3*, *Mest*, *Mfap3l*, *Mfi2*, *Mgarp*, *Micall2*, *Minpp1*, *Mst1*, *Nav2*, *Nckap5*, *Ncoa1*, *Nsg2*, *Nsmaf*, *Ogn*, *Omd*, *Osbp2*, *P2ry2*, *P4ha2*, *Panx3*, *Papss2*, *Pdgfrl*, *Plagl1*, *Ppp1r3g*, *Prkcz*, *Proser2*, *Prr9*, *Psors1c2*, ***Ptx3*** ^5^, *Rab11fip5*, *Rab3ip*, *Ralgps2*, *Ramp3*, *Rarres1*, *Rasgef1a*, *Rasgef1b*, *Rasl11b*, *Rell1*, *Rgl1*, *Rhd*, *Rnf39*, *Rrp1b*, *Scin*, *Sdc3*, *Serpina9*, *Slc13a4*, *Slc26a7*, *Slc29a4*, *Slc2a13*, *Slc7a8*, *Smad6*, *Smad7*, ***Smoc2*** ^6^, *Soga1*, *Sostdc1*, *Spon2*, *Spsb4*, *Stc2*, *Tacr1*, *Tagap*, *Tceal3*, *Tceal6*, *Tmem213*, *Tmem229b*, *Tmem64*, *Trim40*, *Trpv4*, ***Vegfa*** ^7^, ***Vwf*** ^8^, *Wdr26*, *Wif1*, *Wwp2*, *Yod1*, *Zfp395*, *Zfp704*
Downregulated*P* < 0.005FC > 2.0*n* = 67Bold, vascular	*1700012B09Rik*, *2810021J22Rik*, *3930401B19Rik*, *4631405J19Rik*, *4921534A09Rik*, *4930431F12Rik*, *4933402N22Rik*, *9530077C05Rik*, *Apol7b*, *Arl14ep*, *Bfsp1*, *Ccdc116*, *Ccdc122*, *Cd3d*, *Cdk5r1*, *Clec7a*, *Crabp1*, *Crisp3*, *Csta*, *Cyp4f14*, *Dnajc19*, *Dtx3l*, *Dut*, *Erbb3*, *Fam216b*, *Fbxl13*, *Gm20186*, *Gm5919*, *Gngt1*, *Gpr180*, *Gpx2*, *Grin2c*, *Gsg1l*, *Gt(ROSA)26Sor*, *Gzma*, *Il1rl1*, *Itgb3bp*, *Kif5b*, *Klhl30*, *Lgi2*, *Lipi*, *Lrdd*, *Mboat1*, ***Ndp*** ^9^, *Ndufa4*, *Nhlrc4*, *Nkain1*, *Oit1*, *Olfr1148*, *Olfr743*, *Orc4*, *Plce1*, *Prl2c5*, *Samd12*, *Serpina1c*, *Serpinb1a*, *Sfrp4*, *Sh3bgr*, *Slc26a3*, *Snhg3*, *Speer3*, *Steap4*, *Tmprss11g*, *Tnfsf11*, *Uty*, *Vmn1r181*

^1^ Xu et al. 2011; ^2^Rubina et al. 2007; ^3^Kane et al. 2008; ^4^Liu et al. 2007; ^5^Leali et al. 2010; ^6^Rocnik et al. 2006; ^7^Ferrara et al. 2003; ^8^Lenting et al. 2012; ^9^Zuercher et al. 2012.

**Figure 3:**
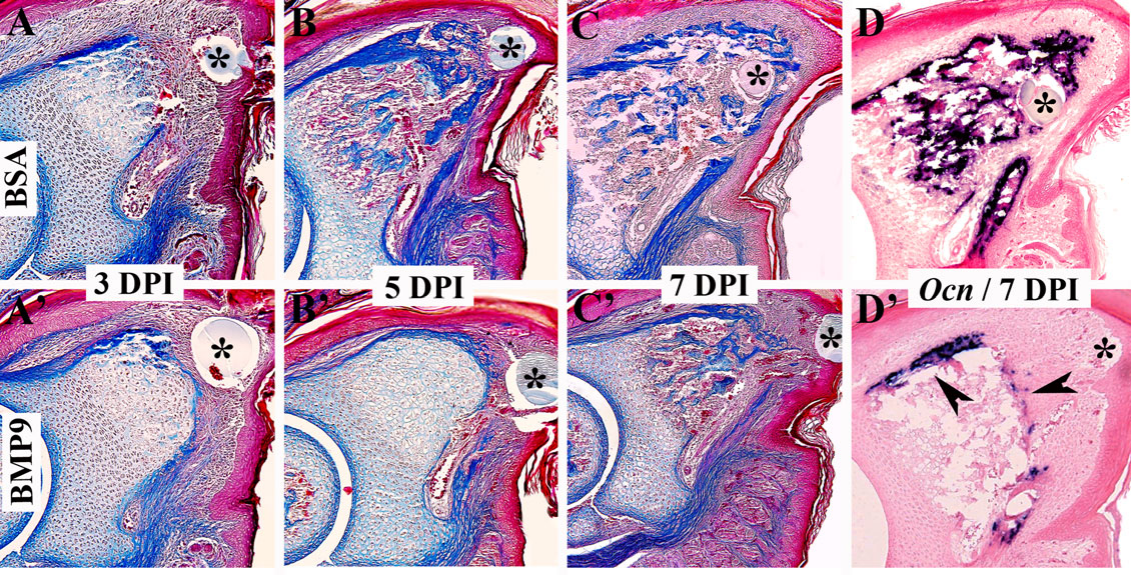
BMP9 treatment delays ossification during regeneration (top is dorsal and proximal is to the left). The microcarrier bead associated with the treatment is indicated with an asterisk. (A)−(D) BSA treated control digits. (A′)−(D′) BMP9 treated digits. (A)−(C), (A′)−(C′) Mallory's triple stained histological sections of amputated digit tips. By 3 DPI the difference between control regenerates (A) and BMP9 treated digit amputations (A′) is subtle. Both accumulate blastema cells distally but ossification of BMP9 treated digit stumps appears to be delayed. By 5 DPI ossification is prominent in the stump and proximal region of control regenerates (B) whereas ossification is clearly delayed following BMP9 treatment (B′). By 7 DPI ossification is occurring throughout the control regenerate (C) whereas ossification is restricted to the stump region of BMP9 treated digits (C′). In situ hybridization to localize transcripts for the osteogenic marker gene, *Osteocalcin* (*Ocn*), validate the histological results. *Ocn* is expressed throughout the control regenerate at 7 DPI (D) whereas transcripts are localized to the periphery (arrowheads) of the stump in BMP9 treated digits (D′).

Along with its effect on angiogenesis, BMP9 is reported to be a potent inducer of mesenchymal stem cell ossification (Lamplot et al. 2013) so another possibility, that is not mutually exclusive, is that BMP9 inhibits the regenerative response by inducing precocious ossification of stump tissues. To address this idea we carried out a stage matched histological analysis of BMP9 treated versus control BSA treated amputations (Fig. 3). At 3 DPI both control and BMP9 treated digits appear to be forming a blastema distally; however, ossification is delayed by BMP9 treatment (Fig. 3A,A′). By 5 DPI the delay in ossification in BMP9 digits compared with control digits involves the entire digit stump and is quite prominent (Fig. 3B,B′). At 7 DPI both BMP9 treated and control digits are undergoing ossification; however, in BMP9 treated digits, ossification does not extend into the distal region of the regenerate and results in a truncated stump (Fig. 3C,C′). The expression of the osteoblast marker gene *Osteocalcin* at 7 DPI clearly identifies both the delay and spatial extent of changes in ossification in BMP9 treated digits (Fig. 3D,D′). Since ossification of the stump eventually occurs (see Fig. 2A), the data show that stump differentiation is delayed while ossification of the regenerate is inhibited by BMP9 treatment. These data show that the inhibition of regeneration by BMP9 is not the result of inducing a precocious ossification response but is linked to an inhibition of ossification that occurs during the redifferentiation phase of regeneration. These data also suggest that digit tip regeneration is not simply an injury induced ossification response by osteoprogenitor cells that are analogous to mesenchymal stem cells.

### Blastema formation is not affected by BMP9 or VEGFa

**Figure 4:**
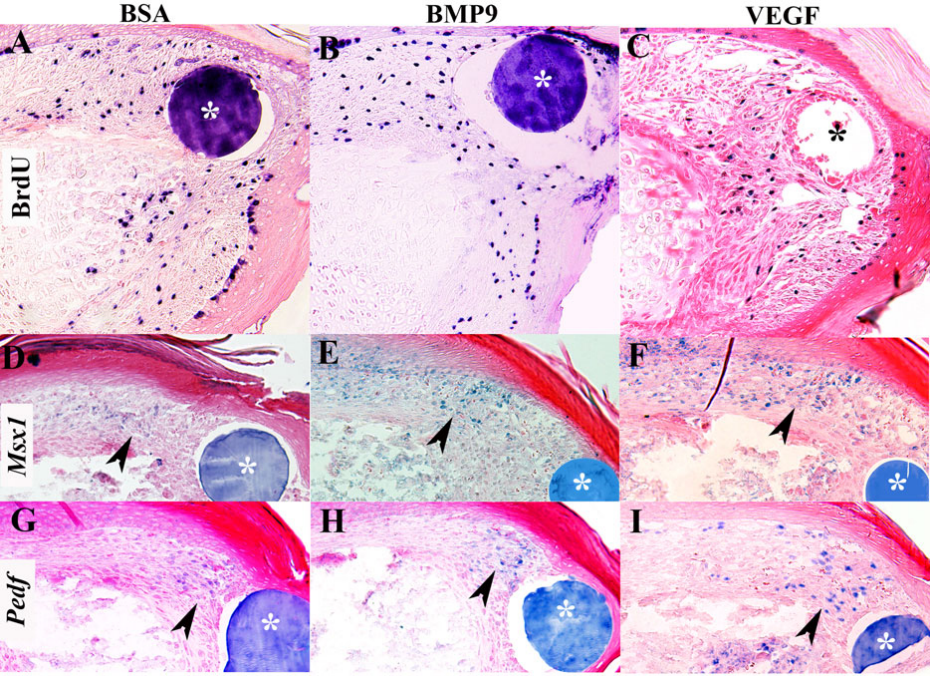
Blastema formation is not influenced by BMP9 or VEGF treatment. The microcarrier bead associated with the treatment is indicated with an asterisk. (A)−(C) BrdU incorporation in digits at 3 DPI shows proliferation in mesenchymal cells of control BSA treated digits (A) and that proliferation is not inhibited by treatment with BMP9 (B) or VEGF (C). (D)−(F) *Msx1* expression was analyzed by section in situ hybridization in digits at 3 DPI. *Msx1* expression (arrowhead) is localized to cells in the dorsal mesenchyme in control BSA treated (D), BMP9 treated (E), and VEGF treated (F) digits indicating that regenerative inhibition is not associated with the suppression of *Msx1* expression. (G)−(I) *Pedf* expression was analyzed by section in situ hybridization in digits at 3 DPI. *Pedf* transcripts (arrowhead) are localized to cells associated with the control BSA treated bead (G), the BMP9 treated bead (H), and the VEGF treated bead (I), indicating that *Pedf* expression is not inhibited by BMP9 or VEGF treatment.

Histological observations suggested that a digit blastema forms following treatment with BMP9. To characterize blastema formation during inhibited regeneration we examined cell proliferation and the expression of established blastema marker genes following BMP9 and VEGF treatment. To explore the simple question of whether the inhibition of digit regeneration is due to an inhibition of cell proliferation, we carried out 5‐bromo‐2'‐deoxyuridine (BrdU) incorporation studies. We observed labeled cells within the distal mesenchyme in BSA treated control, BMP9 treated, and VEGF treated amputations at 3 DPI (Fig. 4A,B,C) indicating that neither BMP9 nor VEGF are inhibitory for cell proliferation and that growth suppression is an unlikely explanation for the inhibition of regeneration. We examined the expression of *Msx1* transcripts to determine whether BMP9 or VEGF was modifying expression of this blastema marker gene. Previous studies have shown that *Msx1* is required for embryonic digit tip regeneration (Han et al. 2003), is transiently upregulated during neonatal blastema formation (Han et al. 2008; Lehoczky et al. 2011) and is induced during BMP2 or BMP7 stimulated digit regeneration (Yu et al. 2010, 2012). The expression domain of *Msx1* is restricted to the dorsal mesenchyme subjacent to the nail in BSA control regenerates at 3 DPI (Fig. 4D), and we find a similar pattern of expression in blastemas following BMP9 or VEGF treatment (Fig. 4E,F). We next examined the expression of *Pedf* in the blastema. *Pedf* expression is associated with blastema formation and has been used as a blastema marker (Muneoka et al. 2008; Yu et al. 2010, 2012) although direct evidence that it plays a role in regeneration is currently lacking. By comparison to endogenous regeneration, *Pedf* expression appeared to be downregulated in control BSA treated digits (Fig. 4G); however, we found no difference between BSA treatment and BMP9 (Fig. 4H) or VEGF (Fig. 4I) treatment. These results indicate that the specific inhibitory effect of BMP9 or VEGF does not involve the inhibition of cell proliferation or a modification of known blastema‐related gene expression. Thus, these studies provide evidence that the inhibitory activity of BMP9 or VEGF on digit tip regeneration does not appear to be linked to an inability of the amputation wound to form a blastema.

### BMP9 and VEGF inhibit regeneration by modulating revascularization

**Figure 5:**
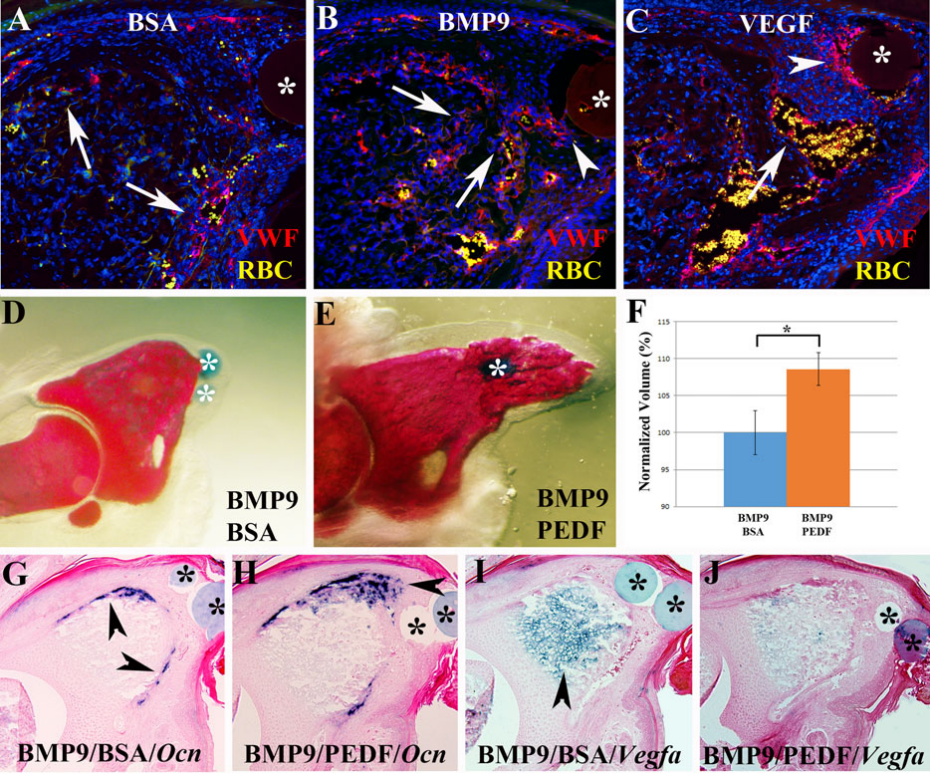
(A)−(C) Revascularization based on immunohistochemical analysis for VWF (red) 7 days after microcarrier bead (*) implantation. Sections were counterstained with DAPI, red blood cells (RBC, yellow) are autofluorescent, distal is to the right. (A) Staining of control BSA treated digits show blood vessels in the periphery of the digit stump (arrows) and sparse staining in the blastema. (B) VWF staining of BMP9 treated digits show blood vessels throughout the digit stump (arrows) and extending into the blastema (arrowhead). (C) After VEGF treatment an extensive vascular response is evident in the digit stump (arrow) and in blastema cells (arrowhead) associated with the bead (*). (D)−(F) The inhibitor activity of BMP9 is rescued following treatment with the anti‐angiogenic factor PEDF. (D) Whole mount skeletal staining shows that the BMP9 inhibited regenerative response is not modified by a control BSA bead implanted 1 day later. (E) BMP9 treatment followed by the implantation of a PEDF bead 1 day later restores the regenerative ability as shown by whole mount skeletal staining of digits at 14 DPI. (F) Bone volume analyzed by microCT is significantly increased by PEDF treatment. Data are normalized to the BMP9 inhibited BSA control digits, *P* < 0.01 (*). (G)−(J) In situ hybridization of control and PEDF rescued regenerates 7 days after implantation. (G) BMP9 inhibited *Ocn* expression (arrowheads) is not modified by BSA control bead implantation. (H) In PEDF rescued BMP9 inhibited digits *Ocn* expression extends distally (arrowhead) into the blastema region. (I) Upregulation of *Vegfa* transcripts (arrowhead) in the stump of BMP9 inhibited digits is not modified by control BSA treatment. (J) In PEDF rescued BMP9 inhibited digits *Vegfa* expression is downregulated and appears similar to untreated regenerates at a similar stage (see Fig. 1C).

One of the known functions of VEGF is to stimulate angiogenesis and we have shown an enhancement of endothelial cells in the blastema after VEGF treatment. To explore the biological effect of BMP9 and VEGF on angiogenesis during later stages of regeneration we carried out immunohistochemical studies using VWF to identify endothelial cells during early redifferentiation (7 DPI). In control BSA treated digits at 7 DPI, VWF staining identified endothelial cells associated with vessels (identified by red blood cell autofluorescence) that were localized along the periphery of the bone stump (Fig. 5A). In BMP9 treated digits, VWF staining identified large vascular beds throughout the entire bone stump (Fig. 5B), correlating with the upregulation of *Vegfa* transcripts induced by BMP9 at this time point (see Fig. 2G). The region of enhanced vascularity extended into the blastema and encompassed cells associated with the BMP9 bead (see arrowhead in Fig. 5B). In VEGF treated digits at 7 DPI, VWF staining was enhanced in the digit stump and as well by blastema cells surrounding the VEGF bead (Fig. 5C). These studies provide evidence that both BMP9 and VEGF treatments cause enhanced revascularization of the regenerating digit stump and differentiating blastema.

We tested the hypothesis that BMP9 inhibition of digit regeneration was mediated by its effect on angiogenesis by carrying out regenerative rescue experiments that relied on the anti‐angiogenic activity of PEDF. PEDF is known to be anti‐angiogenic and in cultured endothelial cells inhibits VEGF activity by modulation of VEGFRII activity (Cai et al. 2011). Since BMP9 does not modify *Pedf* expression but stimulated *Vegfa* expression, we reasoned that inhibiting the activity of VEGF with PEDF treatment would rescue the BMP9 inhibited regeneration response. Digits were treated with BMP9 beads at 4 DPA and subsequently treated with PEDF beads 24 h later when enhanced *Vegfa* expression is first observed. Control studies in which BSA beads were implanted into BMP9 treated digits had no rescuing activity and resulted in stunted regenerates as previously described (Fig. 5D). Conversely, PEDF treatment was able to rescue the regenerative response in 83% of the treated digits (20/24), thus counteracting the inhibitory effect of BMP9 (Fig. 5E). MicroCT analysis of digit bone volumes showed a statistically significant increase in bone volume over BMP9 inhibited control regenerates (*P* < 0.01), thus reinforcing the anatomical rescue response (Fig. 5F). We next analyzed the expression of *Osteocalcin* and found that the inhibited expression resulting from BMP9 treatment (Fig. 5G) was rescued by PEDF treatment at 7 DPI (Fig. 5H). Finally, we explored whether BMP9 induced *Vegfa* expression was modified in PEDF rescued regenerates. BMP9/BSA treated digits showed an enhanced level of *Vegfa* expression associated with the digit stump at 7 DPI (Fig. 5I), whereas the level of *Vegfa* transcripts was considerably depressed in PEDF rescued regenerates (Fig. 5J). Based on these results we conclude that BMP9 inhibits digit regeneration by inducing angiogenic activity via upregulation of *Vegfa* expression. Overall, these studies provide strong evidence that reduced angiogenesis during wound healing and blastema formation is critical for a successful regenerative response and that one important function of endogenously expressed *Pedf* during digit regeneration is to modulate the revascularization response.

## Discussion

The regenerative capacity following amputation of the terminal phalanx in mice represents a unique model for uncovering cellular and molecular mechanisms controlling mammalian regeneration (Han et al. 2008; Muneoka et al. 2008). The regeneration response is characterized by delayed wound closure, extensive erosion of stump bone, blastema formation and redifferentiation of amputated bone by direct ossification (Han et al. 2008; Fernando et al. 2011). The transcriptional repressor *Msx1* and BMP signaling is required for digit tip regeneration (Han et al. 2008; Yu et al. 2010) and ectopic treatment with some BMPs (BMP2, BMP7) during the wound healing phase can induce a regenerative response (Yu et al. 2010, 2012). Thus, this mammalian regeneration model has been used for both loss of function (inhibited regeneration) as well as gain of function (induced regeneration) studies. In the current investigation we studied the role of angiogenesis in the regeneration response by treating the regenerating digit with three known modulators of angiogenesis: VEGF, BMP9 and PEDF. Our results show that VEGF and BMP9 are potent inhibitors of regeneration when extrinsically delivered to the amputation wound. Since BMP9 induces precocious *Vegfa* expression in the amputated stump the data suggest a model in which precocious production of VEGF induces angiogenesis and is inhibitory for a mammalian regeneration response. This model is supported by experiments in which the anti‐angiogenic factor PEDF inhibits BMP9 induced *Vegfa* expression and rescues BMP9 inhibited regeneration. These studies provide loss of function and subsequent gain of function evidence that the regulation of angiogenesis during wound healing following amputation is critical for a successful regenerative response in mammals.

### Angiogenesis and regeneration

Angiogenesis has been extensively studied during mammalian wound healing and is generally thought to play a positive role in wound repair (Wietecha et al. 2013); thus it is curious that enhancing angiogenesis during amputation wound healing actually inhibits the regeneration response. While this conclusion might appear counterintuitive, it is important to keep in mind that mammalian wound healing is a repair process that is typically not regenerative (Seifert et al. 2012). Most mammalian wound healing models are associated with the formation of a highly proliferative and angiogenic granulation tissue that replaces the blood clot within the wound bed (Kawasumi et al. 2013). The granulation tissue is composed of multiple cell types, including fibroblasts, macrophages and endothelial cells, interspersed within a loose extracellular matrix that mediates wound contraction and eventually becomes remodeled into scar tissue (Greaves et al. 2013). The granulation tissue counterpart in a regenerating digit amputation wound is the blastema, a proliferative collection of multiple cell types including fibroblasts, macrophages and endothelial cells, in addition to osteoprogenitor cells (Fernando et al. 2011; Lehoczky et al. 2011; Wu et al. 2013; Simkin & Muneoka, unpublished). The environment within granulation tissue and, as well, the blastema is hypoxic (Wietecha et al. 2013; Sammarco et al. 2014), yet while granulation tissue is characterized by high levels of the *Vegfa*, a known downstream target of HIF1α (Semenza 2010), we fail to observe a similar upregulation during blastema formation. These findings show that hypoxia‐induced angiogenesis that is characteristic of the typical mammalian wound healing response is inhibited during blastema formation and regeneration. This coupled with the demonstration that VEGF is a potent inhibitor of the regenerative response lead to the conclusion that regulation of *Vegfa* expression during wound healing is an important target for modifying mammalian regeneration. Since *Pedf* is expressed in association with the regeneration response we suggest that PEDF functions to suppress VEGF activity thereby helping to create a regeneration‐permissive wound environment.

Mammals in general are considered non‐regenerative and there is speculation that regenerative ability has been lost through selection for other physiological processes that are critical for survival but not permissive for regeneration. For example the acquisition of a complex immune system is hypothesized to have selected against regenerative responses to injury in favor of the need to control the activity of infectious agents (Mescher & Neff 2005). In this study, we provide evidence that supports this general concept but focuses on the role of angiogenesis in wound repair versus regeneration. The evidence suggests that the angiogenesis associated with wound healing has evolved in mammals to restore tissue function following injury. Since early angiogenesis is inhibitory for regeneration, the selection for rapid angiogenesis is predicted to secondarily result in a reduced regenerative response. On the other hand, we previously provided evidence that the regenerating digit tip is an example of an evolved regenerative response (Han et al. 2008; Muneoka et al. 2008; Fernando et al. 2011). This would presumably involve the modification of a non‐regenerative amputation wound into a regeneration‐permissive wound that supports blastema formation. The conclusion that rapid angiogenesis is inhibitory for regeneration indicates that such an evolved regenerative response requires the production of an anti‐angiogenic agent to establish an avascular wound bed. Following digit amputation, the expression of *Pedf* during early wound healing and blastema formation, the lack of expression at non‐regenerative amputations, and the induction of *Pedf* in association with BMP induced digit regeneration (Muneoka et al. 2008; Yu et al. 2010, 2012) all support the view that modification of *Pedf* expression following amputation played a critical role in the evolution of mammalian regeneration. The recent report that *Pedf* expression is reduced early in the healing response of full thickness wounds in mice also supports this conclusion (Chen & DiPietro 2014). Understanding how endogenous regeneration evolved in mammals will provide important clues for addressing regenerative competency in humans.

### Inhibition of regeneration

In amphibians, regeneration is inhibited by local irradiation (Maden & Wallace 1976), by denervation (Singer 1952), by modifying wound closure (Tassava & Mescher 1975), and more recently by inhibiting the inflammatory response (Godwin et al. 2013) or by modifying key metabolic pathways (Love et al. 2013). In all of these examples regeneration is stunted early in the process: blastema formation is inhibited and the regenerative response is terminated. Regenerative failure with blastema formation has been described in experiments that modify positional information during limb regeneration (Bryant 1976), and these studies have been interpreted in terms of position‐specific cell−cell interactions necessary for regeneration (Bryant et al. 1981). It is generally accepted that intercellular signaling is required for the development as well as regeneration of the limb and a number of essential signaling pathways have been identified (Simon & Tanaka 2013). Many of these pathways involve the secretion of a ligand that traverses intercellular space to interact with a receptor on a neighboring cell. Thus, it is reasonable to suppose that the physical environment influencing the intercellular space must be conducive for the transfer of secreted signals. By comparison, the difference between a vascularized versus an avascular microenvironment is predicted to be quite dramatic with the avascular environment being more conducive for intercellular signaling. With this in mind, we suggest that one way blastema avascularity creates a regeneration‐permissive microenvironment is by allowing intercellular signaling necessary for regeneration. Such signaling might include position‐specific interactions as well as signaling important for the orderly differentiation of progenitor cells.

An alternative, but not mutually exclusive, possibility is that flooding the wound bed with a differentiation promoting factor such as VEGF or BMP9 might negatively impact regeneration by redirecting available stem progenitor cells to differentiate disproportional numbers of specific cell types. For example, VEGF might drive excessive numbers of stem progenitor cells into an endothelial lineage, or BMP9 might drive excessive osteogenesis. Indeed, one reason for testing BMP9 in digit regeneration was based on similarities to BMP2 enhancement of mesenchymal progenitor cell osteogenesis (Lamplot et al. 2013). However, our studies very clearly differentiate the effect of BMP9 as an inhibitor of regeneration from inductive activity of BMP2 (Yu et al. 2010), and show that BMP9 delays rather than enhances digit ossification. In the case of VEGF treatment, endothelial cell lineage studies show a lineage restriction during digit regeneration (Rinkevich et al. 2011), and it is well established that VEGF stimulates endothelial cell proliferation (Ferrara et al. 2003); thus it seems unlikely that redirecting a stem progenitor cell population is responsible for the observed regenerative failure. However, we cannot exclude the possibility that a proportional distribution of cell types within the blastema is inhibitory for the regenerative response.

### BMP signaling and regeneration

BMP9 is a member of the BMP family of secreted signaling molecules in the transforming growth factor β superfamily (Hogan 1996). Studies using transduced mesenchymal progenitor cell lines show that BMP9 promotes osteoblast differentiation in vitro and ectopic bone formation following engraftment in vivo (Lamplot et al. 2013). This activity is similar to other better studied BMPs such as BMP2 and BMP7. Like BMP2, BMP9 has been shown to signal via the canonical SMAD pathway in studies on endothelial cells (Larrivee et al. 2012), whereas BMP9 has been shown to activate alternative (non‐canonical) pathways in other cell types (Zhao et al. 2012). Similarly, BMP9 has been shown to stimulate proliferation of some cell types (Song et al. 1995; Ploemacher et al. 1999), but inhibits proliferation in others (Wang et al. 2011). These findings highlight the diversity of BMP9 activity and point to the importance of understanding cell type specific responses to this systemically available factor. In previous studies on digit regeneration we have shown that both BMP2 and BMP7 induce skeletal regeneration (Yu et al. 2010, 2012); thus BMP9's inhibitory influence on digit tip regeneration highlights a novel aspect of BMP signaling. While our findings link BMP9's inhibitory effect on regeneration to the modulation of VEGF induced angiogenesis, the context‐dependent nature of BMP9's activities on different cell types precludes generalizing this effect to other regeneration models.

## Materials and Methods

### Digit amputation and bead implantation

In this study, we purchased pregnant outbred CD1 mice from Charles River Laboratory (Wilmington, MA). Distal amputations of the terminal phalanx (P3) were carried out on postnatal day 3 (PN3) as previously described (Han et al. 2008; Yu et al. 2010). On PN7 (4 DPA) a single microcarrier bead was implanted between the wound epithelium and digit stump of each regenerating digit tip as previously described (Yu et al. 2010; Simkin et al. 2013). For PEDF rescue experiments a second microcarrier bead was implanted on PN8 (1 DPI). Affi‐Gel Blue Gel beads (150–200 μm in diameter, Bio‐Rad, Hercules, CA) were pre‐selected and soaked in different concentrations of growth factor diluted in phosphate‐buffered saline (PBS) with 0.1% BSA. Growth factors used included recombinant human BMP9, recombinant mouse VEGF‐A (VEGF) and recombinant human Serpin F1/PEDF (R&D Systems, Minneapolis, MN). Control beads were soaked in a solution containing 0.1% BSA in PBS. VEGF and BMP9 were tested at soaking concentrations ranging from 10 to 500 ng/μL. PEDF rescue experiments were carried out using beads soaked in a concentration of 1.0 μg/μL. Estimates of the in vivo concentrations of introduced factors based on bead size, wet and dry weight, soaking concentration, and tissue volume at the implantation site were all above traditionally accepted physiological range at the time of implantation. All experimental procedures involving animals were approved by the Institutional Animal Care and Use Committee of Tulane University Health Sciences Center.

### Skeletal analysis

The skeletal structure of the terminal phalangeal element was analyzed after whole mount staining with Alizarin Red S (Han et al. 2008) 2–6 weeks after bead implantation. Samples were subsequently imaged using microCT for three‐dimensional rendering and quantification of bone volume as previously described (Sammarco et al. 2014). MicroCT images were acquired using a VivaCT 40 (Scanco Medical AG, Brüttisellen, Switzerland) at 1000 projections per 180° with a voxel resolution of 10 μm^3^, and energy and intensity settings of 45 kV and 177 μA. Integration time for capturing the projections was set to 200 ms using continuous rotation. Images were segmented using the BoneJ 28 (version 1.2.1) Optimize Threshold Plugin for ImageJ (version1.48c) (Doube et al. 2010). Bone volume was measured using the BoneJ Volume Fraction Plugin for Image J and quantified as bone volume/total volume (percent BV/TV) where total volume was equal to the average volume of control digits. Statistical significance was based on *t*‐test analysis with *P* < 0.01. Three‐dimensional renderings of the microCT scans were created using the 3D viewer plugin for ImageJ.

### Histology, in situ hybridization and immunohistochemistry

For histological and immunohistochemistry studies samples were fixed overnight with Z‐Fix (Anatech Ltd, Battlle Creek, MI) at room temperature and then treated with Decalcifier I (Surgipath) for 2 h. Digits was processed, embedded in paraffin and sectioned at 4–5 μm. For histological studies sections were stained with Mallory's triple stain as previously described (Han et al. 2008). For immunohistochemistry studies sections were stained with an endothelial cell specific polyclonal rabbit anti‐human von Willebrand factor (VWF, Dako; diluted 1:1500) and an Alexa Fluor® 568 goat anti‐rabbit IgG secondary antibody (Invitrogen; diluted 1:500).Immunohistochemical staining was performed following the manufacturer's suggested protocol.

Cell proliferation studies were carried out by using the BrdU labeling and detection kit II (Roche) following the manufacturer's suggested protocol as previously described (Yu et al. 2012). For in situ hybridization studies digits were fixed in 4% paraformaldehyde in PBS at 4°C overnight. Digits were processed for paraffin embedding and sectioned at 8–10 μm. Section in situ hybridization was performed as previously described (Han et al. 2008). The following antisense riboprobes were generated by using the Digoxigenin‐UTP transcription labeling according to the manufacturer's introduction (Roche): *Bmp9* (707 bp), *Vegfa* (748 bp), *Vegfb* (8000 bp), *Osteocalcin* (300 bp), *Msx1* (800 bp) and *Pedf* (*Serpinf1*, 1.0 kb).

### Microarray and real‐time RT‐PCR

The digit samples were collected, snap frozen and embedded in OCT. Digits were sectioned along the proximal−distal axis at 50 μm and central sections were collected and stored in RNAlater®‐Ice (Ambion) at −20°C overnight. Five to six central sections per digit were dissected to remove most of the epidermis by cutting along lines shown in Figure S2A. Isolated blastema tissue from 8–10 digits was collected and RNA was extracted using RNeasy Plus Micro Kit (Qiagen) according to the manufacturer's instruction. RNA was analyzed using an Agilent 2100 Bioanalyzer. Samples with an RNA concentration above 6 ng/μL and a RNA integrity number greater than 7.0 were used for microarray analyses. Eight total microarrays were analyzed, four for BMP9 treatment and four for BSA treatment.

Microarray analysis was performed using the Agilent Mouse Gene Expression 8×60K G3 microarray format (G 4852A) (Agilent Technologies). 10 ng of total RNA was amplified and labeled using the one‐color (Cy3) labeling kit as recommended by the manufacturer. For each sample, 600 ng of Cy3 labeled cRNAs were fragmented and hybridized for 17 h in a rotating hybridization oven at 65°C. Slides were washed and then scanned with an Agilent Scanner, and data were obtained using the Agilent Feature Extraction software (v9.5) according to the manufacturer's instruction (Agilent Technologies). The resulting data were analyzed using GeneSpring bioinformatics software (version 12.6), implementing the unpaired unequal variance (Welch) *t*‐test to determine significance. Genes identified as differentially expressed between BMP9 and BSA treatments are based on a 2.0 or greater fold change and a *P* value <0.005.

Microarray results were validated using quantitative‐PCR using residual RNA samples and the SuperScript® III Platinum® One‐Step Quantitative RT‐PCR System (Invitrogen, Life Technologies). Real‐time quantitative‐PCR was performed with an Eppendorf Realplex2 system with the following probes of Taq‐Man gene expression assays (Invitrogen, Life Technologies): fibroblast growth factor receptor 3 (*Fgfr3* Mm00433294_m1), fibroblast growth factor 18 (*Fgf18* Mm00433286_m1), SPARC related modular calcium binding 2 (*Smoc2* Mm00491553_m10), vascular endothelial growth factor A (*Vegfa* Mm01281449_m1), and fibromodulin (*Fmod* Mm00491215_m1). Mouse Rpl12 Endogenous Control (Life Technologies) was used as an internal control and to normalize expression levels.

## Supporting information

Additional Supporting Information may be found in the online version of this article at the publisher's website:

**Figure S1**. Regeneration is inhibited following VEGF or BMP9 treatment.
**Figure S2**. Microarray analysis and real‐time quantitative PCR validation of digit regeneration.
