# Supplementary material for: Angiogenesis is inhibitory for mammalian digit regeneration
Source: Regeneration (Oxf). 2014 Oct 12;1(3):33–46. doi: 10.1002/reg2.24 (PMC4895301; doi:10.1002/reg2.24)
Supplement: Supplementary file 1 — Figure S1. Regeneration is inhibited following VEGF or BMP9 treatment. Figure S2. Microarray analysis and real‐time quantitative PCR validation of digit regeneration. [file REG2-1-33-s002.pdf]

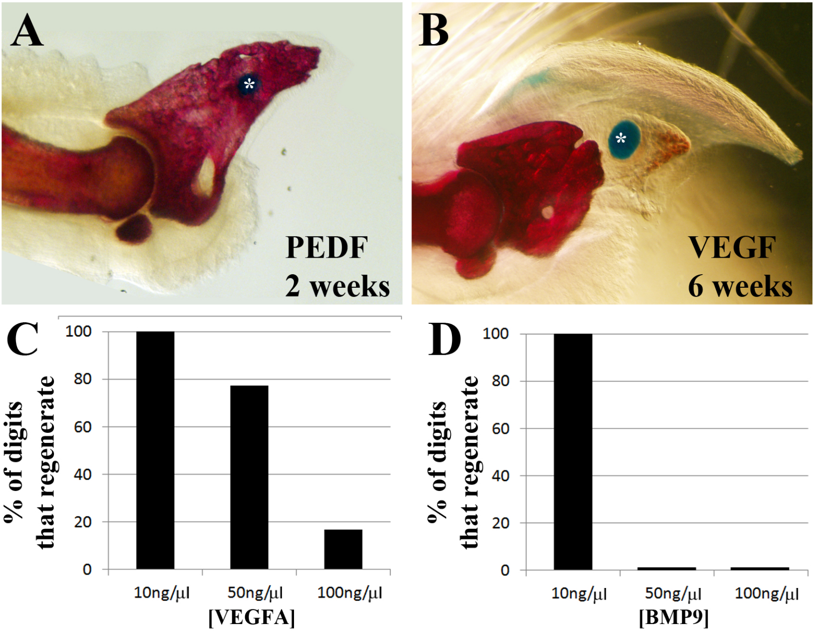

Supplemental Figure 1 – Regeneration is inhibited following VEGF or BMP9 treatment. A) PEDF treatment does not inhibit the regenerative response. B) VEGF treated digits exhibit an inhibition of regeneration 6 weeks after treatment. Microcarrier bead associated with the treatment is indicated with an * in A and B. C) VEGF inhibits regeneration in a dose-dependent manner. All digits regenerated when treated with VEGF at 10 ng//µl, 77% regenerated following treatment with 50 ng//µl and only 17% of digits regenerated when treated with 100 ng/µl. D) BMP9 inhibits regeneration in a dose-dependent manner. All digits regenerated when treated with BMP9 at 10 ng//µl whereas digit regeneration was completely inhibited after treatment with 50 or 100 ng/µl.

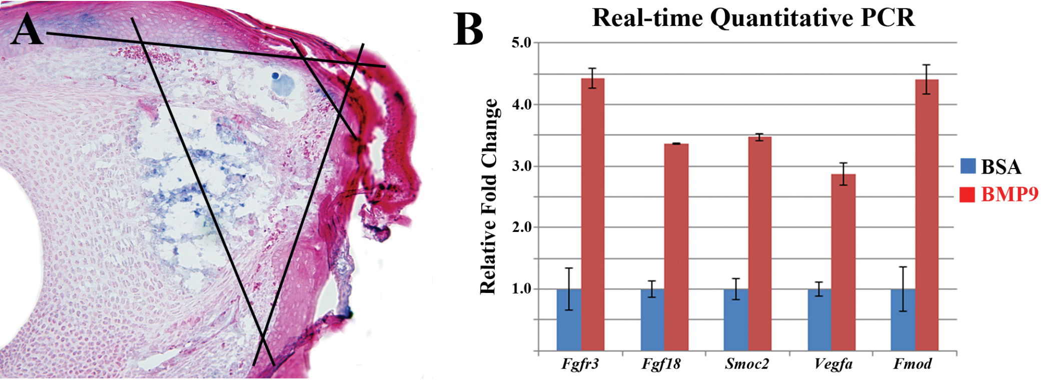

Supplemental Figure 2 – Microarray analysis and real-time quantitative PCR validation of digit regeneration. A) To illustrate how tissue was collected for microarray analysis, an in situ hybridization section stained for Vegfa transcripts 24 hours after BMP9 bead implantation is shown. Digits were snap frozen and embedded in OCT. 50 µm cryosections were cut parallel to the proximal-distal axis of the digit and central sections were collected. Using a scalpel the epidermis was trimmed following cut lines shown and the distal region of the regenerate was isolated with a proximal cut. Tissue was collected from 8-10 regenerates for each BSA or BMP9 treated microarray. B) To validate the microarray data, 5 genes that were up-regulated were selected for further analysis using real-time quantitative PCR. All five genes were found to be up-regulated by comparison to BSA controls.
